# dearseq: a variance component score test for RNA-seq differential analysis that effectively controls the false discovery rate

**DOI:** 10.1093/nargab/lqaa093

**Published:** 2020-11-19

**Authors:** Marine Gauthier, Denis Agniel, Rodolphe Thiébaut, Boris P Hejblum

**Affiliations:** INRIA SISTM, INSERM Bordeaux Population Health Research Center, University of Bordeaux, F-33000 Bordeaux, France; Vaccine Research Institute, F-94000 Créteil, France; Rand Corporation, Santa Monica, CA 90401, USA; Harvard Medical School, Boston, MA 02115, USA; INRIA SISTM, INSERM Bordeaux Population Health Research Center, University of Bordeaux, F-33000 Bordeaux, France; Vaccine Research Institute, F-94000 Créteil, France; CHU de Bordeaux, F-33000 Bordeaux, France; INRIA SISTM, INSERM Bordeaux Population Health Research Center, University of Bordeaux, F-33000 Bordeaux, France; Vaccine Research Institute, F-94000 Créteil, France

## Abstract

RNA-seq studies are growing in size and popularity. We provide evidence that the most commonly used methods for differential expression analysis (DEA) may yield too many false positive results in some situations. We present dearseq, a new method for DEA that controls the false discovery rate (FDR) without making any assumption about the true distribution of RNA-seq data. We show that dearseq controls the FDR while maintaining strong statistical power compared to the most popular methods. We demonstrate this behavior with mathematical proofs, simulations and a real data set from a study of tuberculosis, where our method produces fewer apparent false positives.

## INTRODUCTION

With the rise of next-generation sequencing technologies that measure gene expression on the scale of the entire genome, RNA-seq differential expression analysis (DEA) has become ubiquitous in many research fields. While numerous approaches have been proposed to perform DEA of RNA-seq data, there is no clear consensus on which method is the most efficient. Three methods stand out as the most commonly used in practice: edgeR (1), DESeq2 (2) and limma-voom (3) (6999, 6856 and 1004 citations, respectively, in PubMed as of 11 December 2019). edgeR and DESeq2 both rely on the assumption that gene counts from RNA-seq measurements follow a negative binomial distribution, and limma-voom is based on a weighted linear model and assumes resulting test statistics follow a normal distribution.

Following long-standing statistical practice, researchers typically attempt to control the probability of finding a gene to be differentially expressed (DE) when the opposite is true in reality (i.e. type I error) at a prespecified level (conventionally 5%). In a high-dimensional context such as gene expression data, the false positive rate or false discovery rate (FDR) (4) has been largely adopted as the target probability to be controlled in exploratory studies. The FDR is the expected proportion of features identified as significant that are actually false positives: for instance, an FDR of 5% implies that among all the genes declared DE, 5% are not DE in reality. Controlling this error rate results in fewer false positives than controlling the per-gene type I error, while not being as restrictive as controlling the probability of any false positive (the family-wise error rate) among all of the potentially thousands of genes.

This control is usually taken for granted and often left out from the benchmarks of DEA methods, while in fact an excessive FDR can be quite problematic. Not controlling the FDR means getting more false positives than expected, which limits the reproducibility of study results. Whole genome DEAs are usually exploratory steps prior to subsequent studies to confirm a gene signature is associated with a particular biological condition. If a majority of the selected genes turn out to be false positives, results may fail to replicate and any downstream health benefits may remain elusive, not to mention the waste of research resources.

When comparing DEA methods, the evaluation of their empirical FDR with respect to the targeted (nominal) level is often overlooked (5–10). Nonetheless, some issues with inflated FDR in DEA have been previously reported in the literature (11–15), but those warnings have made little apparent impact on DEA practices.

Inflation of the empirical FDR in DEA can have numerous causes, from inadequate preprocessing of the data to violations of the DEA method’s underlying assumptions. In particular, edgeR, DESeq2 and limma-voom make potentially strong distributional assumptions on RNA-seq data. This type of model-based inference may be required when RNA-seq studies include only a small number of observations. However, these methods’ parametric assumptions are not typically verifiable in practice. Any deviation from the hypothesized distribution of test statistics will translate into ill-behaved *P*-values and therefore uncontrolled FDR. FDR control rests upon the entire distribution of *P*-values being uniform under the null hypothesis *H*_0_ (i.e. for genes that are truly not DE).

So even a slight deviation from strict type I error control can have dramatic consequences on the empirical FDR. In addition, even if type I error were controlled at say 5%, nonuniformity in the *P*-value distribution under the null hypothesis could lead to failure to control the type I error at lower levels (such as 1% or lower) and/or failure to control the FDR. Larger sample sizes do not always solve issues with *P*-value distributions and FDR control arising from violation of modeling assumptions, and can sometimes even exacerbate the problem of misspecification and its consequences. As sequencing costs keep falling and study sample sizes are increasing, this issue needs to be addressed in large sample sizes as well.

Here, we propose dearseq, a new method to perform DEA that effectively controls the FDR, regardless of the distribution of the underlying data. dearseq is a robust approach that uses a variance component score test and relies on nonparametric regression to account for the intrinsic heteroscedasticity of RNA-seq data.

In the ‘Results’ section, we compare the performance of dearseq to the three most popular state-of-the-art methods for DEA: edgeR, DESeq2 and limma-voom. We demonstrate that dearseq enforces strict control of type I error and FDR while maintaining good statistical power in a realistic and extensive simulation study where knowing the ground truth facilitates benchmarking the properties of the different methods. We also present a comparative re-analysis of a real-world tuberculosis (TB) data set from Singhania *et al.* (16) studying apparent false positives identified by the leading DEA methods compared to dearseq. dearseq can efficiently identify the genes whose expression is significantly associated with one or several factors of interest in complex experimental designs (including longitudinal observations) from RNA-seq data while providing robust control of FDR. dearseq is freely available as an R package on the Bioconductor library.

## MATERIALS AND METHODS

The general objective of DEA is to identify genes whose expression is significantly associated with a set of clinically relevant characteristics. dearseq is a new DEA framework based on a variance component score test (17–19), a flexible and powerful test that requires few assumptions to guarantee rigorous control of type I and false discovery error rates. The method can be adapted to various experimental designs (comparisons of multiple biological conditions, repeated or longitudinal measurements, integrated supervision by several biomarkers at once). It builds upon recent methodological developments for the analysis of genomic data (19–21). Variance component tests offer the speed and simplicity of classical score tests, but potentially gain statistical power by using many fewer degrees of freedom and have been shown to have locally optimal power in some situations (22).

The dearseq method comprises three steps (with an optional initial normalization):

1. (optional) normalize gene expression across samples;
2. estimate the mean–variance relationship through a local linear regression borrowing information across all genes;
3. test each gene;
4. apply a multiple-testing correction controlling the FDR, such as the Benjamini–Hochberg procedure

### Model specification

Let *G* be the total number of observed genes. Let $r_{i}^g$ be the raw count of the *g*th gene for the *i*th sample (*i* = 1, …, *n*) . Consider now $y_{i}^g$ the normalized gene expression (any normalization can be used such as log counts per million (cpm) values; see Supplementary File S1 for more details). To build a variance component score test statistic, we rely on the following working linear model for each gene *g*:

(1) $$\begin{equation*} y_{i}^g = \alpha _{0}^g + X_i \boldsymbol{\alpha }^g + \Phi _i \boldsymbol{\beta }^g + \varepsilon _{i}^g ,\end{equation*}$$

where $\varepsilon _{i}^g \sim N(0,\sigma _{i}^g)$, $\alpha _{0}^g$ is the intercept, *X*_*i*_ is a vector of covariates to adjust for and Φ_*i*_ contains the variables for DEA, such as disease status, treatment arm or other clinical characteristics that are to be associated with gene expression. The parameter of interest is $\boldsymbol{\beta }^g$: if $\boldsymbol{\beta }^g \ne \boldsymbol{0}$, then the gene is DE. The variance of the residuals $\varepsilon _{i}^g$ depends on *i* to model the heteroscedasticity inherent to RNA-seq data.

Note that the model presented above is very flexible, and can be easily extended to grouped (e.g. repeated or longitudinal) data to take into account heterogeneity between individuals by adding random effects (see Supplementary File S1 for more details).

### Estimation of the mean–variance relationship

Because of their count nature, RNA-seq data are intrinsically heteroscedastic. We model this mean–variance relationship through $\sigma ^g_i$. But obviously, this individual variance cannot be estimated from a single observation. Instead, we adopt a strategy similar to voom and we gather information across all *G* genes through a local linear regression (23) to estimate $\widehat{\sigma}_i^g$ in a rigorous and principled manner (see Supplementary File S1 for more details).

### Variance component score test statistic estimation

According to the working model (1), a gene is DE and has its expression associated with the variable(s) of interest in Φ if $\boldsymbol{\beta }^g \ne 0$. dearseq thus tests the following null hypothesis for each gene *g*:

(2) $$\begin{equation*} H_0^g: \boldsymbol{\beta }^g = 0 .\end{equation*}$$

The associated variance component score test statistic can be written as $Q^g={\boldsymbol{q}^g}^T \boldsymbol{q}^g$, with

(3) $$\begin{equation*} {\boldsymbol{q}^g}^T = n^{-1/2}\sum _{i=1}^n (y_{i}^g-\mu _{i}^g) {\sigma _i^g}^{-1} \Phi _{i} ,\end{equation*}$$

where *μ*_*i*_ is the conditional mean expression given the covariates *X*_*i*_ (see Supplementary File S1 for more details). Again, this formula can easily generalize to more complex experimental designs such as grouped measurements by incorporating a random-effects covariance matrix (see Supplementary File S1 for more details).

Because this is a score test, we only need to estimate $\widehat{Q}^g$ under the null hypothesis of no differential expression. We estimate $\widehat{\mu }_i$ through ordinary least squares. Finally, since there are a total of *G* tests (with *G* often >10 000), it is absolutely necessary to correct for multiple-testing correction, for instance, by using the Benjamini–Hochberg procedure.

### Asymptotic and permutation tests

The asymptotic distribution of the test statistic *Q* can be shown to be a mixture of $\chi _1^2$ random variables:

(4) $$\begin{equation*} Q \rightarrow \sum _{l=1}^{n_i} a_l \chi _1^2, \end{equation*}$$

where the mixing coefficients *a*_*l*_ depend on the covariance of $\boldsymbol{q}$ (see Supplementary File S1 for details). This asymptotic result rests solely upon the central limit theorem, and this is why dearseq is particularly robust to misspecification: the distribution of *Q* is the same whether model (1) holds or not. Therefore, the type I error and the FDR are controlled as long as the central limit theorem is in action (meaning *n* is large enough).

One advantage of using a variance component score test over a regular score test is the gain in statistical power that comes from exploiting the correlation among $\boldsymbol{\beta }^g$ coefficients to potentially reduce the degrees of freedom of the test. Another advantage is its flexibility that can accommodate random effects in the model to test mixed hypotheses (see Supplementary File S1 for details).

To overcome the shortcomings of this asymptotic test in small samples, we propose to use a permutation test using the same statistic *Q*. Since we are in a multiple-testing setting, it is of the utmost importance to carefully compute the associated *P*-values (24) before applying the Benjamini–Hochberg correction. Finally, in order to preserve statistical power, we use Phipson and Smyth’s correction to account for random permutations (see Supplementary File S1).

### Availability of data and materials

dearseq is freely available on Bioconductor at https://bioconductor.org/packages/release/bioc/html/dearseq.html. The sequence data set from the Singhania *et al.* TB study analyzed in this article is accessible from the NCBI GEO database with the primary accession code GSE107991. The code used to analyze the data set and the results are available from the GitHub repository (https://github.com/borishejblum/dearseq).

### Software versions

All computations were run under R v3.6.1 using DESeq2 package v1.25.11, edgeR package v3.27.13, limma package v3.41.16 and dearseq package v0.99.8.

## RESULTS

### Synthetic simulation study

As highlighted by both Conesa *et al.* (25) and Assefa *et al.* (15), engaging in realistic yet clear simulations of gene expression is difficult. One has to find the right balance between the controlled settings necessary to know the ground truth and the realism necessary to be convincing that the results would translate in real-world analyses. In an attempt to cover as broad a spectrum as possible, we present a performance evaluation of our methods under four data-generating scenarios: (i) a negative binomial parametric assumption for RNA-seq data; (ii) a highly nonlinear model designed to violate most modeling assumptions; (iii) a resampling from SEQC data (26) with truncated Gaussian noise; and (iv) a data-driven negative binomial parametric assumption. Simulations (i) and (ii) were designed to drastically depart from the models used for all methods, whereas simulations (iii) and (iv) aim to be more realistic. Scenario (i) may be more favorable to edgeR and DESeq2 as it relies on their parametric assumption of a negative binomial distribution for RNA-seq count data. Scenario (ii) may be unfavorable for all three compared methods (edgeR, DESeq2 and limma-voom) since it features a high degree of nonlinearity, deviating from any assumed model. Scenario (iii) is likely the most realistic of the three because it relies only on resampling real RNA-seq samples from the SEQC study (26), similarly to what was done by Germain *et al.* (13). A multivariate truncated Gaussian noise (using the estimated covariance structure across the observed genes) was added to enable the generation of larger sample sizes while preserving the count nature of the data. Scenario (iv) relies on a negative binomial distribution whose parameters are estimated using data from Singhania *et al.* (16) in order to avoid using arbitrary settings. Like scenario (i), it favors both edgeR and DESeq2 as they both rely on the negative binomial distribution assumption for the counts.

We simulated 1000 synthetic data sets at different sample sizes using each one of these four scenarios. For scenarios (i) and (ii), 0.5% of genes were generated as truly DE, while the remaining 99.5% were not DE, among 10 000 genes. For scenario (iii), since it is based on resampling from homogeneous samples, it was impossible to induce truly DE genes without making further parametric assumptions (which would have made the scenario less realistic). For this reason, in scenario (iii), FDR corresponded to the probability of finding any genes to be DE. In scenario (iv), 5% of the genes were generated as truly DE, while the remaining 95% were not DE, among 10 000 genes. Details of the data-generating mechanisms are provided in Supplementary File S1.

**Figure 1.**
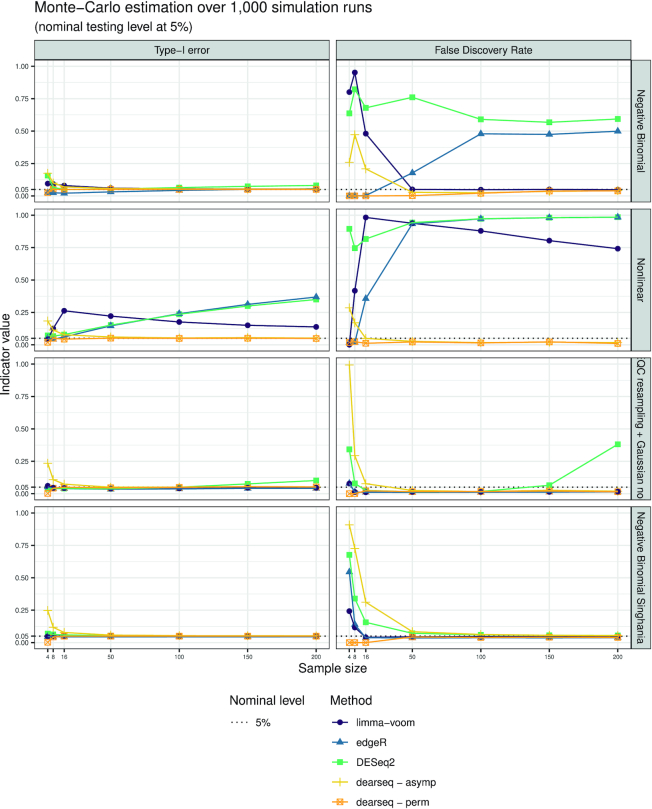
Type I error and FDR curves for each DEA method with increasing sample sizes. In each setting (negative binomial, nonlinear, SEQC data resampling and data-driven negative binomial), the type I error is computed as the number of significant genes among the true negative, and the FDR as the average number of false positives among the genes declared DE.

**Figure 2.**
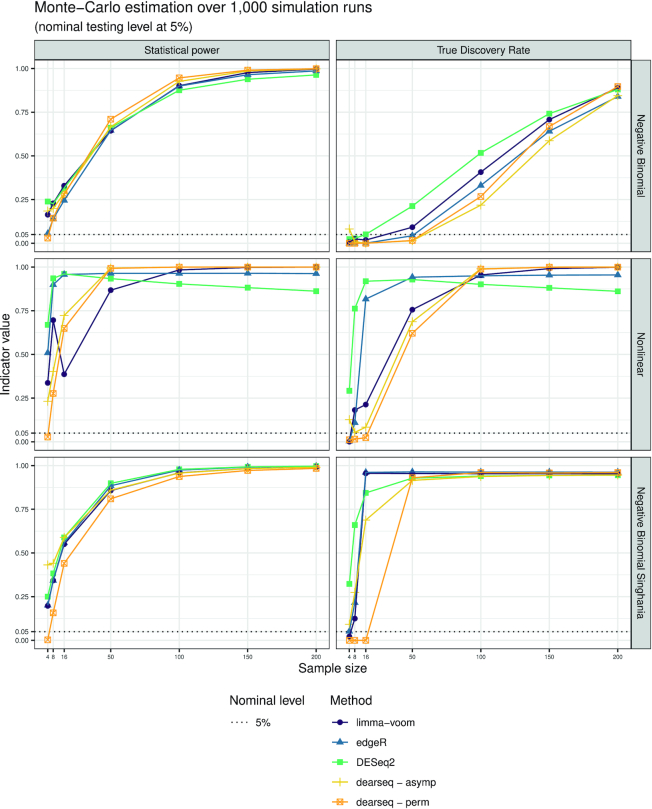
Power and TDR curves for each DEA method with increasing sample sizes. Because SEQC data resampling only generates nonsignificant genes, this setting does not allow to estimate statistical power or TDR.

We evaluated the four methods (the leading methods and dearseq) in terms of type I error control and statistical power, as well as in terms of FDR and true discovery rate (TDR) after Benjamini–Hochberg (4) correction for multiple testing. Throughout, we used a targeted control rate for the FDR at a nominal level of 5%. Figure 1 presents the Monte Carlo estimation over the 1000 simulations in each of the four scenarios for both the type I error and the FDR according to increasing sample sizes (from 4 to 200 samples). Figure 2 presents the results of the first two scenarios and the data-driven negative binomial one for both the statistical power and the TDR. For all edgeR, DESeq2 and limma-voom analyses, we used the default values and followed the guidelines from their respective online user guides. The code executed for all four methods is provided in Supplementary File S2.

In Figure 1, dearseq exhibited good control of both type I error and FDR in all four scenarios, as soon as asymptotic convergence was reached (between 16 and 50 samples depending on the scenarios). To accommodate small sample sizes, we have also developed a permutation-based version of dearseq, which always controlled type I error and FDR, regardless of the sample size. edgeR appeared to control the type I error in scenarios (i), (iii) and (iv) onward, but exhibits slightly inflated type I error for large sample sizes in scenario (i) (from 100 samples). This was much more visible for the FDR, which edgeR failed to control as soon as the sample size rose above 50. In the nonlinear model (ii), neither the type I error nor the FDR is controlled by edgeR. limma-voom exhibited good control of both the type I error and FDR as long as its linear hypothesis was not violated [i.e. not scenario (ii)] and the sample size was large enough (between 8 and 50 samples depending on the scenario). Finally, DESeq2 failed to control either the type I error or the FDR in scenarios (i), (ii) and (iii), with its problems worsening as the sample size increased. In scenario (iv), DESeq2, limma-voom and edgeR exhibited a high FDR at small sample sizes, while the permutation-based version of dearseq controlled the FDR for both small and large sample sizes.

Figure 2 shows that this robust control of type I error and FDR from dearseq does not come at a price of reduced statistical power (or TDR, its multiple-testing correction equivalent). Interestingly, the permutation approach also exhibits good statistical power (regarding competing approaches, interpreting statistical power when the type I error is not controlled would be dubious).

### Real data set

In a recent paper, Singhania *et al.* identified a 373-gene signature of active TB from RNA-seq data (16). TB is a disease caused by a bacterium called *Mycobacterium tuberculosis*. Bacteria typically infect the lungs, but they can also affect other parts of the body. TB can remain in a quiescent state called latent TB infection (LTBI), where the patient is infected but has no clinical, bacteriological or radiological signs of the disease. Participants to this study were recruited from several medical institutes in London, UK [see (27) for a detailed description]. All participants were aged over 18 years. Active TB patients were confirmed by laboratory isolation of *M. tuberculosis* on mycobacterial culture of a respiratory specimen, while latent TB patients were characterized by a positive tuberculin-skin test (TST) together with a positive result using an *M. tuberculosis* antigen-specific IFN-γ release assay (IGRA). Healthy control participants were recruited from volunteers at the National Institute for Medical Research (Mill Hill, London, UK) and were negative to both TST and IGRA. In total, 54 participants were included, of whom 21 were active TB patients, 21 were LTBI patients and 12 were healthy controls.

**Figure 3.**
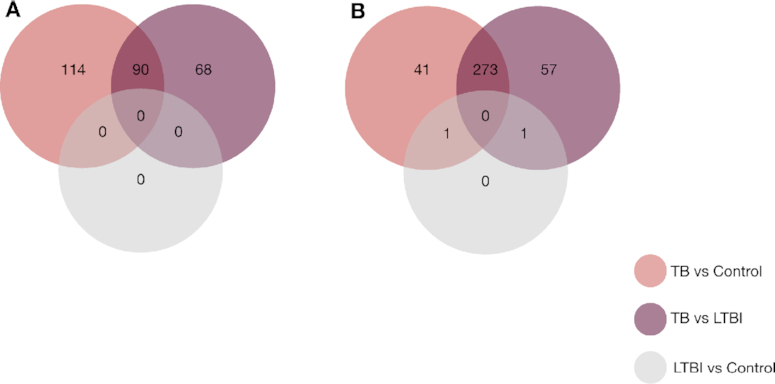
Venn diagram showing overlap of DE genes using dearseq and the original edgeR signature among the three comparisons performed. (**A**) Venn diagram showing the results of the three DEA using dearseq. Note that no DE gene was found with our method comparing the LTBI group and the control group, unlike edgeR that found two such genes to be DE. (**B**) Venn diagram showing the results of the DEA using edgeR (Singhania *et al.*).

The signature was derived by contrasting active TB patients on the one hand against healthy individuals (Control) or those with a latent infection (LTBI) on the other hand (see Figure 3). Their original analysis applied edgeR to their Berry London RNA-seq data, which included 14 150 normalized gene counts measured across 54 samples after preprocessing (see Singhania *et al.* or Supplementary File S1 for more information on this preprocessing) available from GEO (GSE107991). In light of our simulation results, we sought to explore whether the signature Singhania *et al.* found using edgeR was likely to contain false positives. We therefore conducted a comparative re-analysis of these data, first comparing DE genes found by dearseq to the original signature of Singhania *et al.* Second, we further compared the results obtained from the other leading methods, DESeq2 and limma-voom.

Following Singhania *et al.*, to be included in the signature a DE gene *g* must have had both (i) an absolute log_2_(fold change) > 1 and (ii) an FDR adjusted *P*-value <0.05 (after correction for multiple testing with the Benjamini–Hochberg procedure). To ensure reproducibility of the numerical values from Singhania *et al.*, the log_2_ fold changes were calculated using edgeR. The signature was then evaluated by its capacity to distinguish between active TB versus all others. In order to quantify the relevance of each selected gene for distinguishing active TB from control and LTBI, we computed two measures of association, the leave-one-out cross-validated Brier score (28) and the marginal *P*-value for the association between the gene and TB status. The Brier score was computed as ${\rm BS}_g = ({1}/{n}) \sum _{i=1}^n(\widehat{\pi }^{{\rm TB}}_{gi}- {1}_{i\in {\rm TB}})^2$. It compares each patient’s TB status to $\widehat{\pi }^{{\rm TB}}_{gi}$, their predicted probability of TB based on the selected gene *g* estimated using leave-one-out cross-validation. A gene with Brier score BS_*g*_ close to 0 is a good predictor of TB, while a gene with Brier score far away from 0 is a poor predictor and potential false positive. Similarly, we compute the marginal *P*-value for each gene from a logistic regression predicting TB status from the gene expression. We estimate the Brier score and *P*-value for each gene separately. We do this rather than a multivariate model including all genes because the presence of a single predictive gene in the multivariate signature would be enough to yield accurate predictions, thus masking the potential false positive genes included in the model.

**Figure 4.**
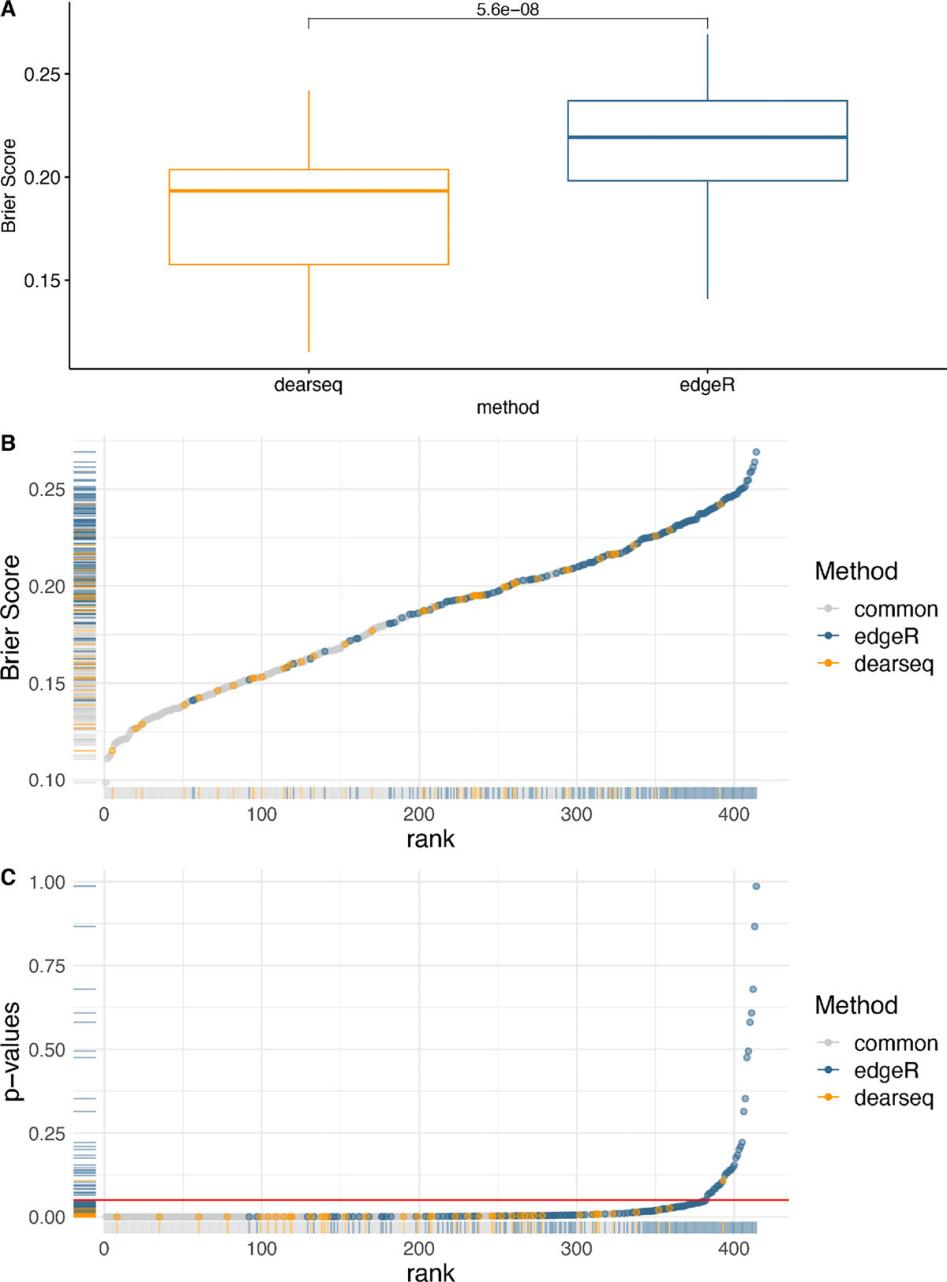
Comparing edgeR-based signature to the signature derived by dearseq. (**A**) Boxplots of the Brier scores of the 41 genes private to dearseq (i.e. not also declared DE by edger) and the 142 genes private to the original edgeR analysis. (**B**) Univariate Brier scores. The blue points correspond to genes found only in the original edgeR signature, the yellow points correspond to genes found only in the dearseq signature and the gray points correspond to genes found in both signatures. (**C**) Marginal *P*-values from a univariate logistic regression combined with a leave-one-out cross-validation for the 40 dearseq-private and the 142 edgeR-private genes. The red line indicates the common 5% *P*-value threshold.

**Figure 5.**
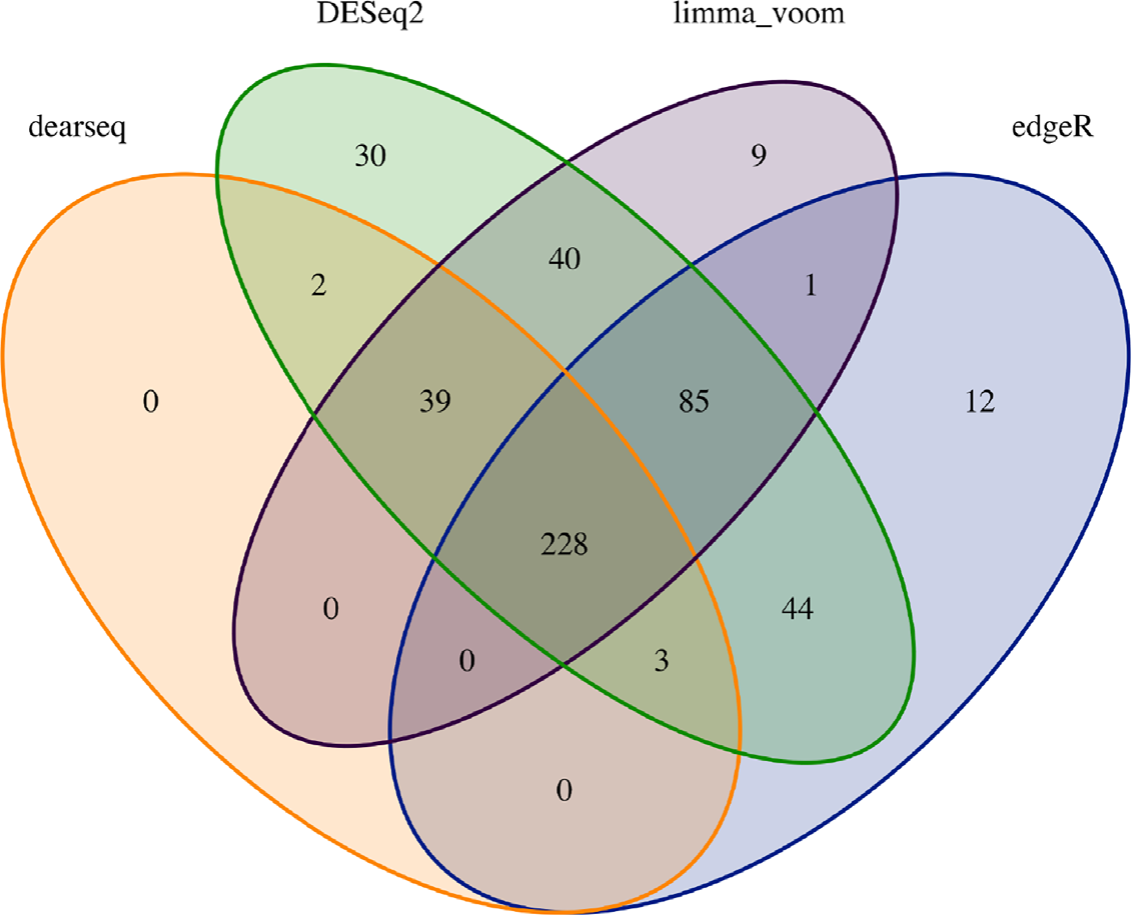
Venn diagram summarizing the different signatures from the four methods. Venn diagram of the genes declared DE by dearseq, DESeq2, limma-voom and edgeR (Singhania *et al.*) under an FDR-adjusted *P*-value of 0.05. None of the genes is found with dearseq only.

Applying the dearseq permutation test (see the ‘Materials and Methods’ section) to the three comparisons originally performed in Singhania *et al.* (TB versus Control, LTBI versus Control and TB versus LTBI) yields a global signature of 272 DE genes (see Figure 3) of which 231 are in common with those found by the original edgeR analysis (see Figure 5). We isolated the genes only identified by dearseq from the genes only identified by edgeR and from the genes in common between the two signatures to further assess the differences between the two results. Comparing the gene-specific Brier scores BS_*g*_ between the two signatures clearly shows that the overwhelming majority of the highest scores (i.e. the lowest predictive abilities) are due to edgeR-private genes (see Figure 4B). Indeed, the univariate Brier scores of the dearseq-private genes have significantly smaller values on average than the edgeR-private genes (according to a *t*-test; see Figure 4). This is further confirmed by the marginal association *P*-values, for which all of the highest values are again from edgeR-private genes, notably all the values above 0.05. Thus, edgeR-private genes are likely false positives, whereas the dearseq-private genes sound more relevant. From a biological point of view, the main pathways concerned by the 142 edgeR-private genes, which are ‘Inhibition of matrix metalloproteinases’, ‘Granulocyte adhesion and diapedesis’ and ‘Inhibition of angiogenesis by TSP1’ using ingenuity pathway analysis, were not directly related to the main pathways observed in the retained 373-gene signature (IFN-inducible genes, B- and T-cell genes) although the interferon signaling pathway was represented by two genes and some upstream regulators. On the contrary, among the 41 dearseq-private genes, HERC5 is upregulated by regulators belonging to IFN signaling pathways including IFNαβ and IFNε (known to be regulated by *M. tuberculosis*). Those results emphasize the better predictive ability of the genes identified by dearseq and highlight the potential false positives arising from edgeR.

**Figure 6.**
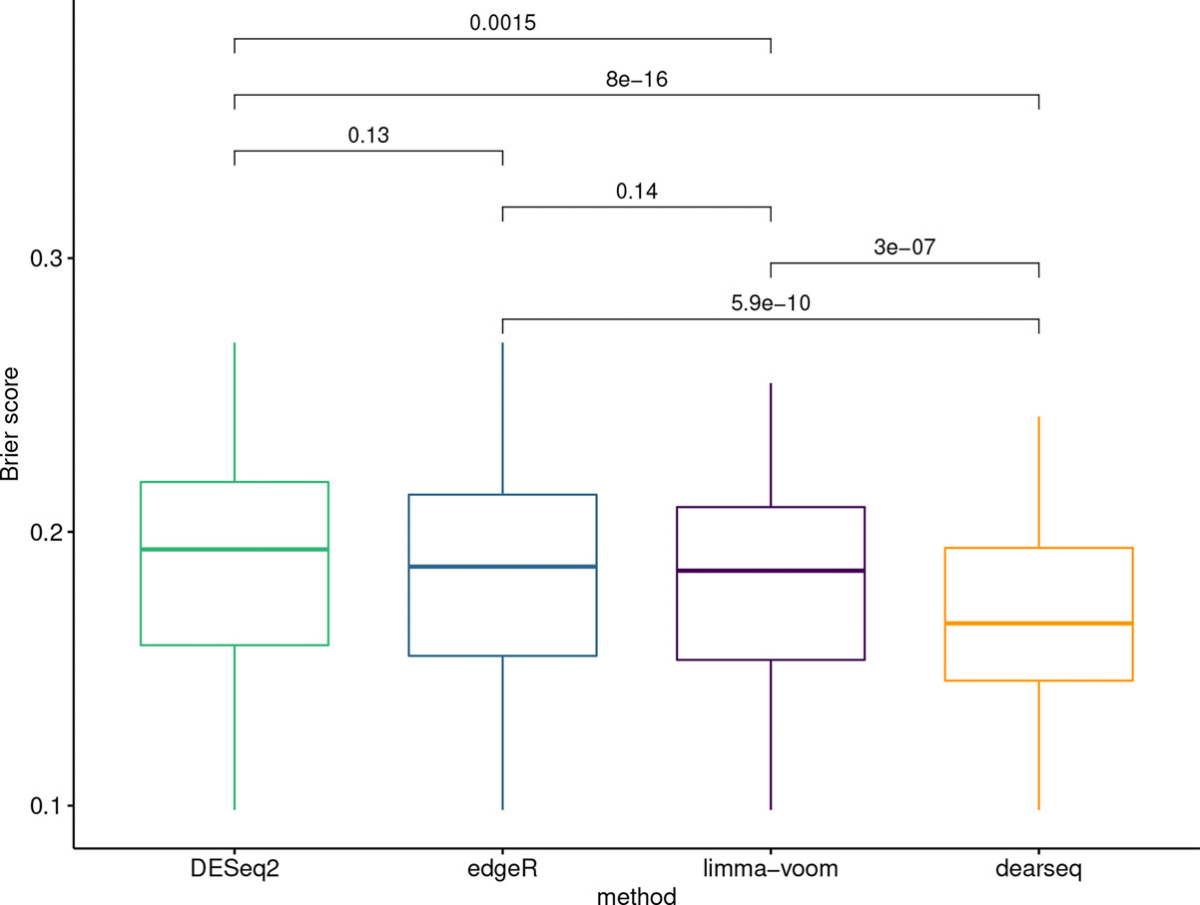
Boxplots of the Brier scores of all the genes declared DE by the four methods. Boxplots of the Brier scores of all the DE genes called by dearseq, DESeq2, limma-voom and edgeR (Singhania *et al.*). The predictions are derived from a logistic regression combined with a leave-one-out cross-validation. Smaller Brier scores are better.

**Figure 7.**
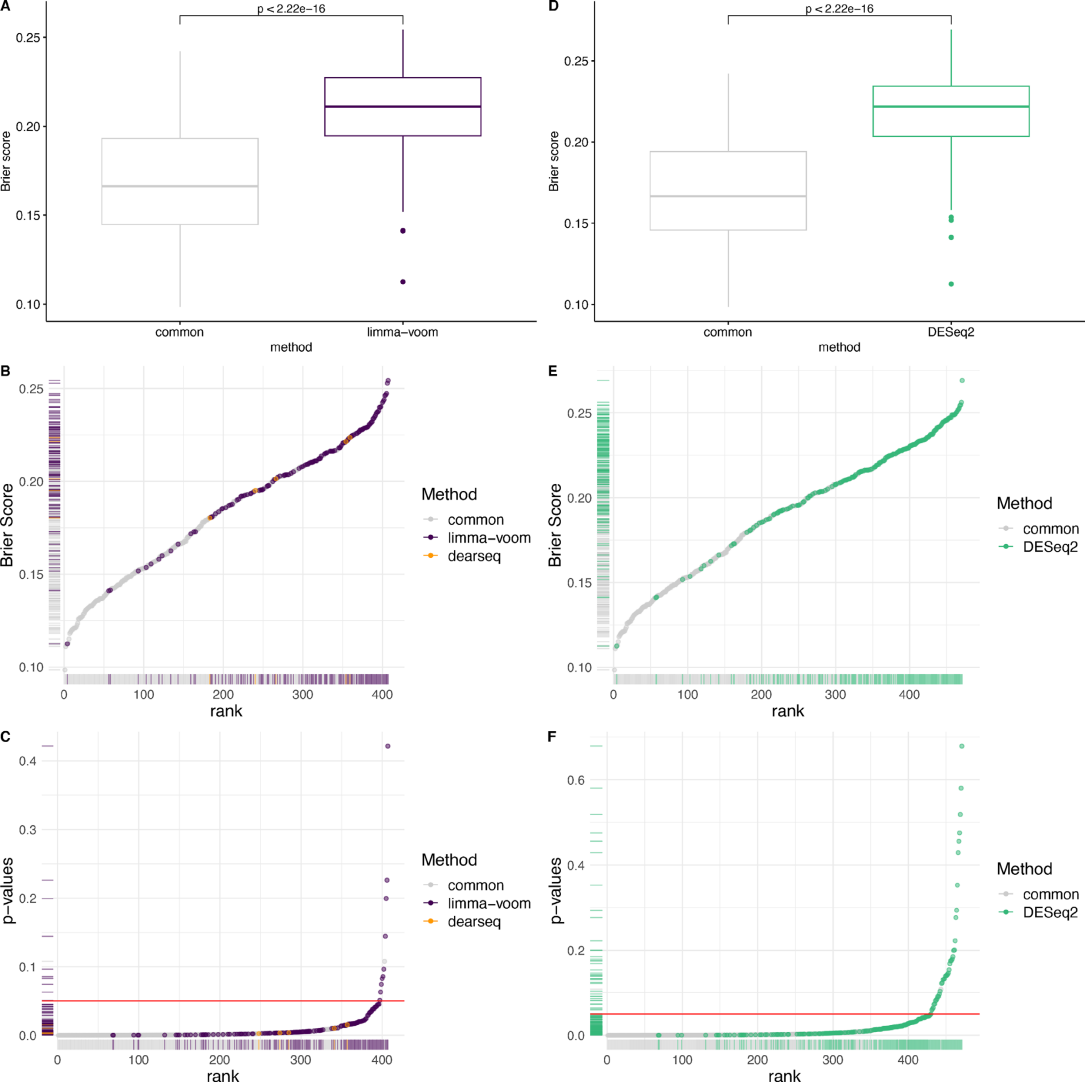
Comparison of the dearseq-derived signature with both the DESeq2- and limma-voom-derived signatures. (**A**) Boxplots of the Brier scores of the DE genes private to limma-voom and the DE genes common to both dearseq and limma-voom. Note that only five genes are identified only by dearseq and not limma-voom. Therefore, we exclude the associated boxplot. (**B**) Univariate Brier scores. The purple points correspond to the DE genes called by limma-voom and the gray points correspond to the genes common with dearseq. (**C**) Marginal *P*-values. (**D**) Boxplots of the Brier scores of the DE genes private to dearseq and the DE genes common to both dearseq and DESeq2. All genes declared DE by dearseq were also declared DE by DESeq2. (**E**) Univariate Brier scores. The green points correspond to the DE genes called by DESeq2 and the gray points correspond to the genes common with dearseq. All genes declared DE by dearseq were also declared DE by DESeq2. (**F**) Marginal *P*-values.

In addition, we performed the same analysis using limma-voom and DESeq2 to further benchmark the performance of dearseq. For DESeq2 and limma-voom, we used the default values following the guidelines from their respective online user guides. For edgeR, we rely on the results directly provided by Singhania *et al.* (16) (see Supplementary File S1 for details and verbatim code in Supplementary File S2). Figure 5 displays the Venn diagram of significantly DE genes across these four analyses. There are 228 genes common across all these tools. Interestingly, all of the 272 genes identified by dearseq are also identified by at least one of the three competing methods (and only 2 genes are identified by less than two other methods—namely only by DESeq2), illustrating that dearseq is less prone to generate false positives. DESeq2 identifies the largest signature comprising 471 genes, including all of the 272 genes identified by dearseq and 360 out of the 373 originally identified by edgeR, while limma-voom identifies 402 genes, among which 267 are in common with dearseq and 314 are in common with edgeR. As can be seen in Figure 6, the dearseq signature has the lowest average Brier score, meaning that most of the additional genes identified by the three competing methods are less predictive of active TB status. Figure 7 strengthens this conclusion by showing again that the limma-voom-private genes are largely over-represented among the highest Brier scores and the highest marginal *P*-values. The same conclusion can be drawn for the DESeq2-private genes.

## DISCUSSION

The proposed method dearseq represents an innovative and flexible approach for performing gene-wise analysis of RNA-seq gene expression measurements with complex design. As demonstrated in our simulation study, edgeR, DESeq2 or limma-voom can all fail to control the type I error and the FDR when the sample size increases, while our method behaves correctly. Moreover, the re-analysis of the London Berry TB data set revealed that the DE genes identified by dearseq are highly predictive of active TB status, while results from the three state-of-the-art methods (including the original edgeR analysis) likely include numerous false positives. While Agniel *et al.* (19) focused on the analysis of longitudinal data and only considered gene set analysis, here we introduce a much broader framework that generalizes the previous mathematical results beyond longitudinal studies and gene set analysis—most notably dearseq allows gene-by-gene analysis and can accommodate many different experimental designs including the usual two (or more) group(s) comparison.

It is important to note that edgeR, DESeq2 or limma-voom will not systematically have inflated FDR. As illustrated by our simulation studies, there are some scenarios in which, for some given sample sizes, they control the FDR adequately. However, we have shown here that this is no guarantee, and in practice it is very difficult to know under which circumstances a data analysis is taking place.

Because dearseq solely relies on the central limit theorem convergence for its asymptotic test to work, it guarantees a control of the FDR without needing any model to hold as long as the sample size is large enough. This contrasts with limma-voom that hinges on weighted least squares and can yield incorrect inference if the heteroskedasticity in the variances is not modeled correctly (29). For lower sample sizes, where convergence is not reached, a robust permutation test can be used instead. Using Phipson and Smyth’s (24) correction, it adequately controls the FDR regardless of the sample size and exhibits good statistical power in our simulation study. An alternative approach to permutation tests would be to use a Bayesian estimate of the posterior *P*-value with either a uniform prior or a Jeffreys’ prior, for instance. Considering *m* the number of permutations and *b* the number of successes, the *P*-value is then equal to ${(b+1)}/{(m+2)}$ or ${(b+1/2)}/{(m+1)}$, which is also never equal to 0. Notice when *m* is large, this ends up very close to the unbiased estimator from Phipson *et al.* Besides, this permutation test introduces a trade-off between the numerical precision (i.e. the number of permutations performed) and the associated computational time. We have undertaken substantial efforts to speed up the implementation of the dearseq R package on Bioconductor, which allows for parallel computing. This leads to reasonable computation times (from a few seconds to no more than a couple of minutes on a laptop) depending on the sample size and the computing power available. If more permutations were deemed essential, one option would be to selectively increase the number of permutations only for the genes where numerical precision is not sufficient to confidently estimate their adjusted *P*-value, thus limiting the computational burden.

Among the three state-of-the-art methods compared here, DESeq2 seems to fail to control the FDR most often. In particular, even under its model assumption of a negative binomial distribution for the data, it can suffer from inflated FDR. This seems counterintuitive as our synthetic data were generated under the negative binomial distribution, and this should benefit DESeq2 and edgeR—since both methods assume this model. As has been noted previously, this behavior can be caused by nonuniformity in the distribution of the *P*-values arising from DESeq2 or edgeR (especially when combined with a multiple-testing correction such as the Benjamini–Hochberg procedure) (12,30–32). In addition, it should be noted that this behavior is not expected to be universal and other parameterizations of the negative binomial generative model could lead to better performance for these methods.

DEA can have numerous preprocessing steps, and the various possibilities can complicate the fair comparison of different methods. Since here our primary goal was to compare to the original edgeR analysis, we used the edgeRpreprocessed data as input to dearseq (dearseq does not rely on a specific preprocessing step and only requires that gene expression measurements are comparable across samples—all preprocessing regarding systematic bias or batch effect must be performed beforehand with any procedure deemed appropriate). For DESeq2 and limma-voom, we used the raw counts. Indeed, both edgeR and DESeq2 assume the input data to be strictly counts (i.e. integers), due to their negative binomial distribution assumption, though edgeR also has some support for so-called non-integer counts. While this seems sensible given the nature of RNA-seq data, recent innovations in RNA-seq alignment methods such as salmon (33) or kallisto (34) return pseudo-counts that are not integers. If the loss of precision is likely not severe when rounding up pseudo-counts, this same limitation prevents the use of already preprocessed (i.e. normalized or transformed) data and forces the DEA practitioner to stick to the specific processing of the methods. In that regard, dearseq is extremely flexible and offers to use either raw or transformed data (the default applies a log-cpm transformation similarly to limma-voom). RNA-seq is subject to various technical biases, and in particular the library size has an important and well-known impact on downstream analysis if ignored. Therefore, it is important to account for library size one way or another, and several RNA-seq data normalizations have been proposed to do so [e.g. TMM, RPKM, FPKM and CPM (35)]. The choice of which normalization to use is often linked to the biological context of an analysis, and thanks to its distribution-free assumptions, dearseq can be paired with any normalization method that is deemed appropriate. Following Law *et al.* (3), we implemented the log_2_ cpm by default.

In addition, these methods have been designed to compare two (or multiple) conditions (several treatment regimens), and are not specifically oriented toward grouped or longitudinal data. Therefore, there is a need in the broader DEA community for a more flexible method. dearseq relies on a general methodology that can easily accommodate more complex designs including gene set analysis while correctly controlling the FDR (19).

We have demonstrated that the three most popular RNA-seq DEA methods may not guarantee control of the number of false positive in their results, even when the sample size increases. To exemplify this problematic behavior, we present extensive simulation studies ranging from realistic resampling of real data to synthetic data generation under the models’ assumptions, as well as a re-analysis of a real-world data set. To offer an alternative solution to DEA practitioners, we have developed dearseq, a new DEA method that uses a variance component score test to provide a robust, powerful and versatile approach to DEA while avoiding the pitfall of FDR inflation exhibited by the current state-of-the-art methods in certain situations. We also benchmarked this new method alongside the three established methods on both the simulations and the real data analysis to illustrate its excellent performance, in terms of both FDR control and statistical power.

These results have important implications for the field, as DEA of RNA-seq data has become widespread. The distributional assumptions and model-based inference inherent to DESeq2, edgeR and limma-voom can underestimate the number of false positives in realistic settings. Users should be aware of the possibility of inflated FDR when using these procedures and should consider the use of dearseq that gives theoretical and empirical control of the FDR without sacrificing its statistical power. Given the results of both our simulations and our real-world data re-analysis, we thus formulate the following recommendations: (i) do not rely on a single DEA method and compare the results across several tools, as this strategy may likely eliminate the majority of false positives; and (ii) for your main analysis, we recommend using dearseq or limma-voom over DESeq2 or edgeR. Indeed, limma-voom appears to control the FDR adequately as long as your sample size is large enough and the model assumptions (in particular, the linearity) are reasonable. On the other hand, dearseq ensures an effective control of the FDR regardless of the sample size (thanks to its permutation test for small sample sizes) and demonstrates good statistical power.

## Supplementary Material

lqaa093_Supplemental_File
